# Stratification of Motor Cortex Excitability to Transcranial Stimulation Uncovers Functional Network Differences in Healthy Older Adults as Revealed by Resting State EEG Functional Coupling in Brain Network

**DOI:** 10.1002/cph4.70136

**Published:** 2026-03-26

**Authors:** Lorenzo Nucci, Federico Frasca, Chiara Pappalettera, Francesca Ginatempo, Nicola Loi, Lucia Ventura, Mohammed Zeroual, Paolo Maria Rossini, Franca Deriu, Fabrizio Vecchio

**Affiliations:** ^1^ Brain Connectivity Laboratory, Department of Neuroscience and Neurorehabilitation IRCCS San Raffaele Roma Rome Italy; ^2^ Department of Theoretical and Applied Sciences eCampus University Novedrate Como Italy; ^3^ Department of Biomedical Sciences University of Sassari Sassari Italy; ^4^ Azienda Ospedaliero Universitaria di Sassari Sassari Italy; ^5^ Unit of Endocrinology, Nutrition and Metabolic Disorders Azienda Ospedaliero Universitaria di Sassari Italy

## Abstract

**Background:**

Understanding the neural organization underlying motor function is essential for explaining individual differences in motor performance and the impact of aging.

**Methods:**

We examined 87 healthy older adults who underwent, in different sessions, resting state electroencephalography (EEG) recordings and Transcranial Magnetic Stimulation (TMS) applied to the hand muscles representation area in the primary motor cortex to elicit Motor Evoked Potentials (MEPs). Furthermore, Mini‐Mental State Examination (MMSE) and handgrip strength evaluations were carried out on subjects. Subjects were split into two groups, Low MEP (L‐MEP) and High MEP (H‐MEP) groups, based on individual MEP amplitude, and age, sex, and education matched. Functional connectivity was analyzed through Magnitude‐Squared Coherence (MSCoh) and Total Coherence (TotCoh) in different frequency bands and brain regions of interest.

**Findings:**

The L‐MEP presented decreased MSCoh in the Alpha 2 and Beta 1 bands, and decreased TotCoh in the Alpha 2 band within the Temporal region as well as in the Beta 1 band across Parietal, Occipital, and Temporal regions. No significant difference in grip strength was found while the MMSE score of L‐MEP group was significantly lower compared to the H‐MEP one. These findings indicate that reduced motor cortex excitability reflects decreased network integration, particularly in regions associated with cognitive and sensorimotor processing. These findings may reflect early neurophysiological vulnerability.

**Conclusions:**

These results underscore the resting state EEG as a non‐invasive, highly sensitive tool monitoring subtle alterations in brain functional networks that may precede clinical symptoms, offering a powerful tool for monitoring and individualized intervention.

## Graphical abstract

Resting‐state EEG revealed that older adults with reduced motor cortex excitability display functional connectivity deficits in cognitive and sensorimotor networks and poorer cognitive performance. These findings suggest that motor excitability indexes early neurophysiological vulnerability and highlight EEG as a sensitive tool for detecting subtle brain network alterations.

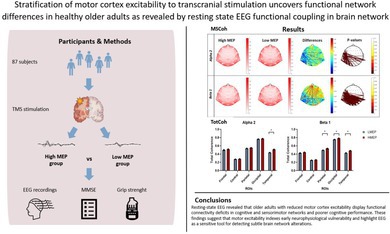

## 1 Introduction

Motor function constitutes a core domain of human physiology, underpinning voluntary movement, goal‐directed behavior and the capacity for independent living. Motor execution depends largely on the structural and functional integrity of cortico‐cortical and cortico‐spinal pathways devoted to movement programming/performance, which facilitate the transmission and integration of motor‐related neural signals (Li et al. 2015). Although motor abilities are shaped by a range of individual and environmental factors, they are subjected to gradual modifications across the lifespan (Kim et al. 2025). Age‐related changes, such as declines in neuromuscular efficiency, muscle strength, cortical excitability, and functional cortical connectivity, may contribute to interindividual variability in motor performance, even in the absence of overt pathology (Rossini et al. 1992). A comprehensive assessment of brain motor circuits' function is therefore critical for elucidating the mechanisms underlying motor control and for identifying functional markers of motor system integrity during aging.

Emerging evidence further supports that the functional properties of motor pathways may be closely associated with cognitive function, highlighting the interconnection between motor and cognitive domains (Buss et al. 2023). Cognitive and motor systems share common neural substrates, and a decline in one domain often mirrors or contributes to decline in the other. Thus, cortico‐cortical and cortico‐spinal integrity may play a dual role in supporting both motor and cognitive health in older age. A valuable tool to investigate the functional status of motor pathways, including motor cortex excitability, in a noninvasive and reproducible manner is Transcranial Magnetic Stimulation (TMS) (Antal et al. 2022; Deriu et al. 2021). This technique allows researchers to explore the excitability, integrity, and plasticity of cortical and cortico‐spinal motor circuits in both health and disease. By delivering brief magnetic pulses over the scalp, TMS can depolarize neurons in the primary motor cortex and elicit Motor Evoked Potentials (MEPs), recorded via surface electromyography (EMG) in corresponding target muscles. MEPs reflect the conduction of neural impulses along central motor pathways and provide an objective measure of their excitability (Ferreri et al. 2017; Ortu et al. 2008). Key parameters commonly analyzed include MEP peak‐to‐peak amplitude, latency, and motor threshold, all of which can be modulated by physiological states, pathological conditions, or experimental interventions. In particular, the magnitude of the muscle response, as measured by the MEP peak‐to‐peak amplitude, is a representation of the number of pyramidal tract neurons (PTNs) depolarized by the TMS pulse (Groppa et al. 2012), with larger MEP amplitudes indicating greater excitability of the PTNs or a greater number of depolarized PTNs, and thus higher levels of excitability (Nitsche and Paulus 2000; Ridding and Rothwell 1997).

An age‐related variability in cortical excitability, and consequently in MEPs amplitude, has been reported in literature, for example when comparing young and old healthy adults. Such variability is also reflected in changes in resting motor threshold (RMT), which tends to increase with age, indicating a general decline in cortico‐spinal excitability in older individuals (Rossini et al. 1992). Furthermore, TMS studies revealed that MEP amplitude is reduced and MEP latency is significantly longer in old than young individuals (Oliviero et al. 2006). MEP differences and variability are also recorded in literature within the same age group, particularly in the elderlies. Sollmann et al. (2017) (Sollmann et al. 2017) associated those variabilities with gender, body height and medications, that are widely common and disparate in the elderly population. Further evidence of intra‐subject MEP variability is reported by Goldsworthy et al. (2021), who noted that while higher MEP amplitudes are typically associated with intact and efficient motor pathways, lower MEPs may indicate reduced central motor activation efficiency, even in the absence of evident pathology. However, the functional significance of this variability remains unclear, especially regarding cortico‐cortical pathways. Indeed, the variability in MEPs may be partly explained by interindividual differences in the organization of neural networks, particularly those most involved in motor function, as reported by Cárdenas‐Morales et al. (2014). To assess this, electroencephalography (EEG) is a key, affordable, non‐invasive, and easy‐to‐set‐up technique that enables the recording of brain electrical activity generated primarily by pyramidal neurons, capturing oscillatory patterns, and allowing to determine functional connectivity between regions that reflect the dynamics of the underlying neural networks. In particular, resting state EEG represents a valuable tool exploring how the organization of functional networks and their connectivity may account for individual differences in motor preparedness and cortico‐cortical excitability (Pellicciari et al. 2013). During resting state, the brain organizes itself to be ready to react to an external solicitation, and its circuits' pattern reflects the readiness of neural networks to engage in upcoming cognitive or motor tasks. An index of intra‐brain communication, that quantifies the grade of synchronization between different brain regions, is represented by EEG coherence. In particular, magnitude‐squared coherence (MSCoh) is a measure of the functional connectivity between two EEG signals recorded at different electrode sites (Núñez et al. 2019; Ruiz‐Gomez et al. 2019). It quantifies the degree of linear correlation between the signals at a specific frequency, indicating how consistently two brain regions oscillate together over time. In this context, EEG coherence between relevant regions could reflect the functional integrity and excitability of the cortico‐cortical system and of the underlying neural network, measuring the synchronization of brain rhythms in these regions. The role of cortical synchronization in motor control has been emphasized by several studies that have investigated the relationship between EEG activity and motor function. These studies often focused on EEG–EMG coherence to assess the coupling between cortical signals and muscular output. For example, Eswari et al. (2025) reported reduced EEG–EMG coherence in older adults compared to younger individuals, suggesting age‐related declines in neural synchrony and motor control efficiency. However, beyond cortico‐muscular coherence, relatively few studies have explored whether intrinsic brain network dynamics, captured through resting state EEG coherence, relate to cortical excitability and motor system responsiveness, as reflected by MEP amplitudes. This represents a notable gap in the current literature, especially considering the growing interest in understanding interindividual variability in motor system responsiveness, even among demographically homogeneous populations.

In the current study, resting state EEG was recorded in 87 healthy older adults to examine functional brain connectivity employing MSCoh. In a separate session, TMS was performed to induce MEPs, and participants were divided into two matched groups, High MEP (H‐MEP) and Low MEP (L‐MEP), based on the median peak‐to‐peak MEP amplitude. Importantly, the study investigates whether changes in resting state functional connectivity are associated with the level of cortico‐spinal excitability, as reflected by MEP amplitude. By directly comparing EEG connectivity patterns between the H‐MEP and L‐MEP groups, we aim to reveal if high responders possess different, more integrated network profiles at rest compared to the low responders' group.

This could support more personalized rehabilitation and neuromodulation strategies, tailored to individual brain connectivity profiles, and improve outcomes in elderly patients with high variability in motor responsiveness.

## 2 Methods

### 2.1 Participants

For this study, a total of 87 subjects (mean age 67.85 ± 0.75 years; range: 52–89 years old; 40 males, 47 females), all right‐handed according to the Oldfield inventory scale, participated in the study.

The experimental procedures were approved by the Local Ethics Committee (Sardinia Ethics Committee Prot. PG/2023/5172, 06/04/2023) and were conducted according to the Declaration of Helsinki. None of the participants had a history and/or current signs/symptoms of neurological and/or psychiatric diseases, and no use of psychotropic drugs (neuroleptics and anticonvulsive medications). Exclusion criteria followed the TMS safety guidelines (Rossi et al. 2020). Additionally, subjects with severe sleep‐related issues were also excluded based on the Pittsburgh Sleep Quality Index (PSQI) (Buysse et al. 1989).

All subjects underwent the Mini‐Mental State Examination (MMSE) (Mazzoni et al. 1992) to assess their cognitive status and assess whether differences in MEP amplitude were associated with changes in cognitive functioning. To evaluate peripheral motor strength, handgrip strength was measured using a handgrip dynamometer (G200, Biometrics LTD, Newport, United Kingdom) over three trials, with one‐minute rest between trials. The best and average results were recorded.

During the recording procedures, participants were seated in a fully relaxed position, on a comfortable armchair, with elbows flexed at 90° and hands in a prone.

### 2.2 Transcranial Magnetic Stimulation (TMS)

TMS was performed using a 70 mm figure‐of‐eight coil connected to a Magstim 200 stimulator (Magstim Co., Whitland, and Dyfed, UK). The optimal stimulation site for the right first dorsal interosseus muscle (FDI) was carefully identified on the skull over the left primary motor cortex and then marked with a soft tip pen on the scalp, to maintain the same coil position throughout the experiments. The handle of the coil pointed posteriorly and laterally at approximately 45° to the interhemispheric line (Rossini et al. 2015).

MEPs were measured from the FDI using surface electromyography, which was recorded using 9‐mm diameter Ag‐AgCl surface electrodes. The active electrode was placed over the muscle belly, the reference electrode over the second finger metacarpophalangeal joint, and the ground electrode over the forearm (Ginatempo et al. 2022). EMG signals were recorded (D360 amplifier, Digitimer Ltd., Welwyn Garden City, UK), amplified (x1,000 times), filtered (bandpass 3–3000 Hz), and sampled at 5 kHz using a 1401 power analog‐to‐digital converter and Signal 6 software (Cambridge Electronic Design, Cambridge, UK).

The MEP amplitude was quantified semi‐automatically as the peak‐to‐peak value between the two largest deflections of opposite polarity. During the session, each participant received 15 suprathreshold TMS pulses at 120% of the individual resting motor threshold (RMT), with an interstimulus interval (ISI) of 5 s to avoid neural habituation (Kujirai et al. 1993; Ziemann et al. 1996). The RMT was defined as the minimum stimulus intensity required to evoke MEPs with an amplitude greater than 50 μV in at least 5 out of 10 consecutive trials in the relaxed state. This procedure allowed for inter‐individual comparability by normalizing stimulation intensity to each participant's cortico‐spinal excitability.

### 2.3 Data Recordings and Pre‐Processing

All participants underwent resting state EEG recordings in an eyes‐closed condition for at least 5 min. The EEG signals were recorded using 64‐channel system (Easycap, GmbH, Brain Products). The electrodes were positioned according to the International 10–20 System. The reference electrode was placed at FCz position, while the ground electrode was placed at Fpz position. Electrode impedance was kept below 5 KΩ and the sampling rate frequency was set up at 1000 Hz.

The EEG data were analyzed in Matlab R 2022b (MathWorks, Natick, MA. 2022) utilizing custom scripts built on the EEGLAB toolbox (Pappalettera et al. 2022).

The EEG data were initially down‐sampled to 512 Hz and band‐pass filtered between 0.2 and 47 Hz utilizing a finite impulse response (FIR) filter. Then, EEG recordings were processed by segmenting the signal into 2‐s epochs, and artifacts, like eye movements, muscle contractions of scalp, and cardiac activity, were detected and removed. This procedure was conducted through an initial visual inspection by an EEG expert, who manually identified and discarded epochs containing artifacts, and then through an automated approach using the Infomax Independent Component Analysis (ICA) algorithm (Aoki et al. 2015; Radüntz et al. 2017).

The ICA algorithm decomposes the signal into statistically independent sources from multi‐channel data, allowing for comprehensive detection, cleaning, and rejection of artefactual components. At the end of the artifact removal procedure, a minimum of 3 min of clean EEG data remained for each participant.

### 2.4 Magnitude‐Squared Coherence (MSCoh)

Magnitude‐Squared Coherence (MSCoh) is a commonly used metric in EEG functional connectivity analysis, quantifying the stability of the phase relationship between two signals over time. It is computed as the squared magnitude of the cross‐spectrum between two EEG signals, divided by the product of their individual power spectral densities, yielding a value between 0 (no coherence) and 1 (perfect coherence). This measure indicates how consistently two brain regions oscillate in unison, offering insight into the level of synchronization and functional communication between cortical areas.

MSCoh is defined as:

$$\text{MSCO}H_{\mathrm{xy}}\left(f\right)=\frac{{\left|G_{\mathrm{xy}}\left(f\right)\right|}^{2}}{G_{\mathrm{xx}}\left(f\right)\cdot G_{\mathrm{xx}}\left(f\right)}$$

where *G*_*xy*_(*f*) is the cross‐spectral density between *x* and *y*, and *G*_*xx*_(*f*) and *G*_*yy*_(*f*) are the auto‐spectral density of x and y respectively. The magnitude of the spectral density is denoted as *|G|* (Fischer et al. 2023). In the present study, coherence was calculated utilizing custom‐made scripts in MATLAB, between each pair of electrodes for each of the following frequency bands: Delta (2–4 Hz), Theta (4–8 Hz), Alpha 1 (8–11 Hz), Alpha 2 (11–13 Hz), Beta 1 (13–20 Hz), Beta 2 (20–30 Hz), and Gamma (30–45 Hz). Topographical maps were also generated to represent the value of the connection between each pair of electrodes for each frequency band. A color scale ranging from white (indicating low coherence) to red (indicating high coherence) was used, providing a concise visualization of the MSCoh.

### 2.5 Total Coherence (TotCoh)

Global functional connectivity, referred to as TotCoh, was assessed for each frequency band by computing the mean coherence across all possible pairs of electrodes. This metric captures the overall level of functional coupling within the EEG signal, offering a comprehensive overview of brain‐wide synchronization (Cacciotti et al. 2024; Musaeus et al. 2019). For each electrode, an individual coherence value was obtained by averaging its coherence with all other electrodes in the montage. These individual values were then averaged to derive the TotCoh, thus representing a global index of functional connectivity based on the collective interaction among all electrode sites. In particular, TotCoh was assessed considering 5 different regions of interest (ROIs): Frontal (Fp1, Fp2, Fpz, AF3, AF4, AF7, AF8, F1, F2, F3, F4, F5, F6, Fz), Central (FC1, FC2, FC3, FC4, FC5, FC6, C1, C2, C3, C4, C5, C6, Cz), Parietal (CP1, CP2, CP3, CP4, CP5, CP6, CPz, P1, P2, P3, P4, P5, P6, P7, P8, Pz), Occipital (O1, O2, Oz, PO3, PO4, PO7, PO8, POz), and Temporal (F7, F8, FT7, FT8, T7, T8, TP7, TP8, TP9, TP10). TotCoh of each ROI was evaluated considering exclusively the coherence of electrode pairs belonging to the region. This approach was intended to examine potential region‐specific patterns of functional connectivity that could be relevant to the interpretation of motor system functioning and MEPs modulation.

### 2.6 Statistical Analysis

Two‐tailed unpaired Student's *t*‐test was conducted to assess statistical differences in MSCoh values between the L‐MEP group and the H‐MEP group across specific frequency bands: Delta, Theta, Alpha 1, Alpha 2, Beta 1, Beta 2 and Gamma. The significance threshold was set at *p* < 0.05. To account for multiple comparisons, the false discovery rate (FDR) correction was applied, yielding an adjusted threshold for each comparison (Yoav Benjamini and Daniel 2001). This method adjusts the *p*‐value threshold according to the rank of each test's *p*‐value and the overall number of comparisons, maintaining the rate of false positives at a specified level (5%).

For each statistically significant frequency band, a composite figure was created consisting of four topographic maps arranged from left to right. The first two maps illustrate the functional connectivity (MSCoh) patterns for the L‐MEP and H‐MEP groups, respectively. The third map illustrates the group differences in connectivity, computed as L‐MEP minus H‐MEP, using a color scale from blue (negative difference), indicating higher H‐MEP group MSCoh values than L‐MEP ones, to red (positive difference), meaning higher L‐MEP group values than H‐MEP, with green indicating no difference. The fourth map highlights only statistically significant differences, corrected for multiple comparisons using False Discovery Rate (FDR). These differences are visualized on a red color scale, where darker shades indicate stronger statistical significance. A color bar accompanies the significance map, ranging from white (non‐significant) to red, with the value *p* = 0.05 centered. Together, these visualizations provide a comprehensive representation of connectivity distributions, group‐level differences, and the spatial localization of statistically significant effects.

Additionally, a three‐way ANOVA was implemented to evaluate the differences in TotCoh values between the factors Group (L‐MEP, H‐MEP), ROI (Frontal, Central, Parietal, Occipital, Temporal), and Band (bands resulted significant in MSCoh analyses). Duncan's post hoc analyses were applied to further investigate significant effects identified by the ANOVA.

Regarding TotCoh, histograms were created to visualize differences between ROIs.

Furthermore, two‐tailed unpaired Student's *t*‐test was performed to assess whether corrected MMSE and Grip strength significantly differed between the L‐MEP and H‐MEP groups, in order to evaluate potential cognitive and motor differences. Data are reported as mean ± standard error (SE).

Finally, a Pearson's correlation analysis was performed between subjects' MEP values and MMSE scores to assess the presence of an association between the two measures.

## 3 Results

### 3.1 Cognitive and Motor Characterization

The 87 participants enrolled were divided into two age‐, sex‐, and education‐matched groups according to the median value of the MEP peak‐to‐peak amplitude of the analyzed sample. Subjects with MEP amplitude lower than the median value (*n* = 42, aged 68.29 ± 0.97 years, 24 females and 18 males) were included in the Low MEP group (L‐MEP), while ones with MEP amplitude higher than the median value (*n* = 45, aged 67.44 ± 1.14 years, 23 females and 22 males) were assigned to the High MEP group (H‐MEP).

Table 1 provides the mean peak‐to‐peak MEP amplitude, mean corrected Mini‐Mental State Examination (cMMSE) score and mean Grip strength value of the two groups, summarizing demographic information. The Student's *t*‐test for the comparisons of corrected MMSE between the L‐MEP and H‐MEP groups reported significant differences, showing a *p*‐value of *p* = 0.037 and a *t*‐value of *t* = −2.134. Regarding the Grip strength, no significant differences between L‐MEP and H‐MEP were assessed. Chi‐squared test was also performed to assess differences in terms of gender between the two groups, and no significant differences emerged (*p* = 0.57).

**TABLE 1:** Demographic, educational, and neurophysiological characteristics of the Low MEP (L‐MEP) and High MEP (H‐MEP) groups. Values are reported as mean ± standard error. Groups were matched for age, sex, and education. MEP = motor evoked potential amplitude; cMMSE = corrected Mini‐Mental State Examination score.

Group	N	Age	Gender (M/F)	Education	MEP (mV)	cMMSE	Grip (kg_f_)
*L‐MEP*	42	68.29 ± 0.97	18/24	12.76 ± 0.49	0.49 ± 0.02	28.89 ± 0.19	27.55 ± 1.50
*H‐MEP*	45	67.44 ± 1.14	22/23	13.51 ± 0.42	1.39 ± 0.11	29.40 ± 0.15	28.43 ± 1.25
Statistics	n/a	*p* = 0.58	*p* = 0.57	*p* = 0.47	*p* < 0.001	*p* = 0.037	*p* = 0.87

Given the observed group difference in MMSE scores, Pearson's correlation analyses were conducted between subjects' MEP amplitudes and MMSE scores to investigate the potential correlation between cortical excitability and global cognitive status. No significant correlation was found between the two measures (*p* = 0.79).

### 3.2 Magnitude Squared Coherence

**FIGURE 1:**
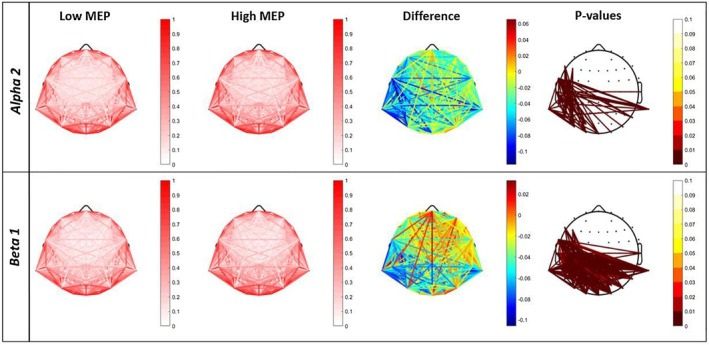
Topographical representation of MSCoh for the Low and High peak‐to‐peak MEP amplitude (L‐MEP and H‐MEP, respectively) groups for significant bands Alpha 2 and Beta 1. Left to right, the first two maps represent the whole set of connection between every possible pair of electrodes of the two groups, with associated colorbar ranging from 0 (white, meaning no coherence) to 1 (red, meaning maximum coherence). The third map represents the differences between L‐MEP and H‐MEP MSCoh (L‐MEP_MSCoh_—H‐MEP_MSCoh_), with associated colorbar representing the value of the difference (blue if negative and red if positive, via yellow if null). The fourth map represents the statistically significant differences, with associated colorbar representing FDR corrected *p*‐values ranging from white (non‐significant result) to red (significant result). Darker colors represent more significant results.

The FDR corrected Student's *t*‐test for the comparisons of MSCoh between the L‐MEP and H‐MEP groups showed significant differences in Alpha 2 and Beta 1 bands (Figure 1).

In Alpha 2 (FDR threshold, *p*_FDR_ = 0.0017) significant differences in MSCoh appeared localized in the left temporal, parietal, and occipital regions. In particular, the L‐MEP group showed significantly lower connectivity compared to the H‐MEP group, suggesting a region‐specific decrease in functional coupling between electrode pairs in these regions. Such a decrease in MSCoh for the L‐MEP group was significantly more widespread and pronounced in the Beta 1 band (FDR threshold, p_FDR_ = 0.0067). In addition to the left temporal, parietal, and occipital regions, the Beta 1 differences extended into central areas, further emphasizing a robust and localized decrease in functional connectivity in individuals with lower MEP.

### 3.3 Total Coherence

The three‐way ANOVA for the evaluation of TotCoh between the factors Group (L‐MEP, H‐MEP), ROI (Frontal, Central, Parietal, Occipital, Temporal), and Band (Alpha 2, Beta 1) showed a significant interaction between Group and ROI (*F*(4,340) = 2.5134, *p* = 0.04150). Duncan's post hoc test demonstrated that the L‐MEP group exhibited lower connectivity in the Temporal region in Alpha 2 (*p* = 4e‐6) and lower connectivity in the Parietal, Occipital, and Temporal regions in the Beta 1 band (*p*_parietal_ = 0.0073, *p*_occipital_ = 0.035, *p*_temporal_ = 0.00018) compared to the H‐MEP group.

**FIGURE 2:**
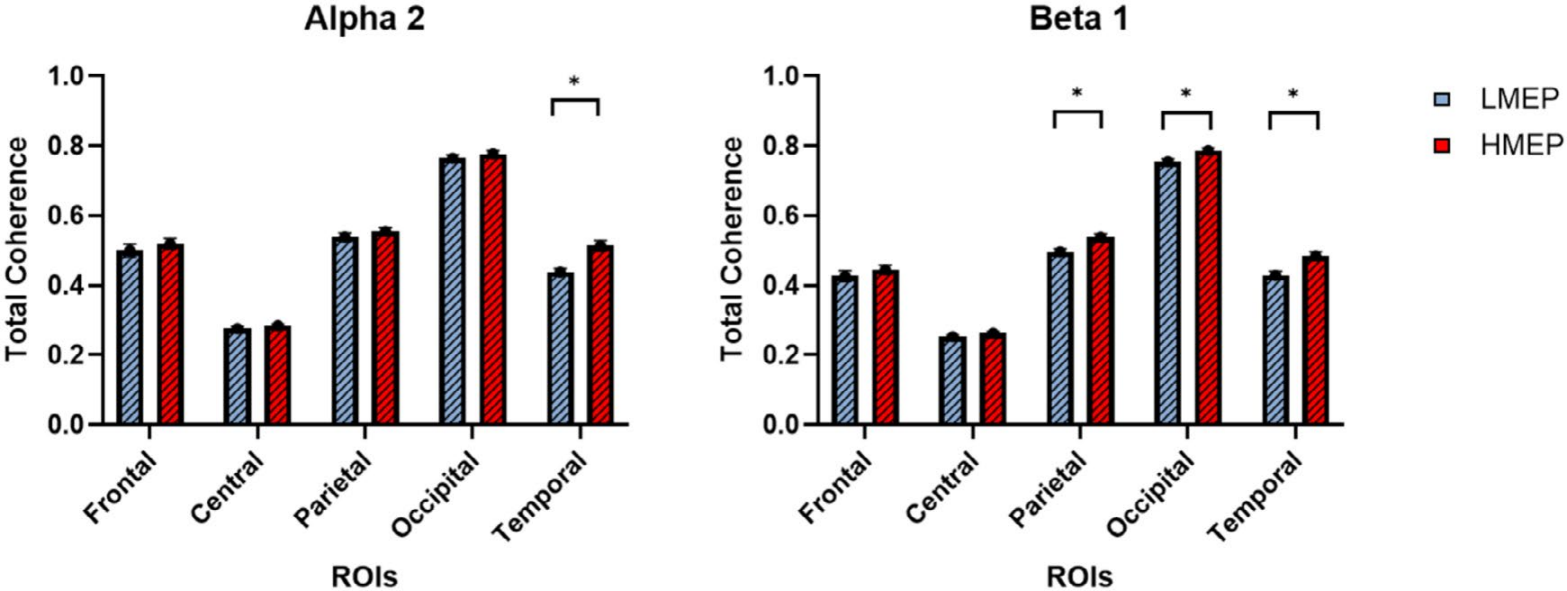
Histograms representing differences between L‐MEP group (blue bars) and H‐MEP group (red bars) TotCoh, for Frontal, Central, Parietal, Occipital and Temporal Region of Interest (ROIs), for Alpha 2 and Beta 1 frequency bands. Significant differences are highlighted with “*”.

A graphical representation of these results is shown in the histogram in Figure 2, containing TotCoh values of L‐MEP (blue bars) and H‐MEP (red bars) groups for Alpha 2 and Beta 1 frequency bands for each ROI (Frontal, Central, Parietal, Occipital, Temporal).

## 4 Discussion

Motor function is a fundamental aspect of human life, enabling independence and autonomy, qualities that become especially crucial in older age, both for the individual and the reality in which they live (Zhang et al. 2024).

The aim of the present study was to investigate differences in resting state neural network organization between older adults with Low peak‐to‐peak MEP amplitude (L‐MEP) and those with High peak‐to‐peak MEP amplitude (H‐MEP). The two groups were formed according to the median value of the entire set of peak‐to‐peak MEP amplitudes and were balanced for sex, age, and education. Neural network differences were assessed in terms of Magnitude‐Squared Coherence (MSCoh) and Total Coherence (TotCoh). In addition, to determine whether the subdivision based on MEP value resulted in cognitive and executive discrepancy, the cognitive and motor status of the two groups was also assessed by investigating the differences in the corrected MMSE and Grip strength.

Regarding the MSCoh, our findings reveal that the L‐MEP group exhibits statistically lower values with respect to the H‐MEP group in Alpha 2 and Beta 1 bands. In the Alpha 2 frequency band, the L‐MEP group showed reduced coherence compared to the H‐MEP group, specifically in the left Temporal, Parietal, and Occipital regions. For the Beta 1 band, the results extended those observed in the Alpha 2 band, with differences also involving the Central region. Considering TotCoh, the L‐MEP group showed statistically lower values in the Alpha 2 band in the Temporal region, while in Beta 1 the L‐MEP group exhibited reduced values in the Parietal, Occipital, and Temporal regions with respect to H‐MEP.

These results, in line with existing literature (Ferreri et al. 2014; Kauv et al. 2025), highlight differences in the organization of resting state neural network between L‐MEP and H‐MEP groups, with L‐MEP showing an overall less connected network. In particular, main differences were assessed in Parietal, Occipital and Temporal regions, reflecting localized changes. The differences in connectivity observed in these regions are supported by various neuroimaging studies showing that the primary motor cortex is not the only region involved in the processing of sensory‐motor information but that Parietal, Temporal and Occipital regions are also functionally integrated (Breveglieri et al. 2021; Pierotti et al. 2025; Tosoni et al. 2015). Furthermore, Alpha 2 and Beta 1 bands are reported in literature as key bands for sensory‐motor signal integration and involved in motor performance, as indicated by Chung et al. (2017) and McFarland et al. (2000) (Chung et al. 2017; McFarland et al. 2000). In particular, the Alpha 2 band has been implicated in top‐down control, attention, and the coordination of large‐scale brain networks (Klimesch 2012).

Results in Alpha 2 band in Temporal region suggest that reduced cortico‐spinal excitability, as reflected by lower MEP amplitudes in the L‐MEP group, may be associated with weaker functional integration in cortical regions beyond the motor system. In particular, the Temporal cortex plays a key role in sensory integration, language, and memory processes (Binder and Desai 2011; Mesulam 1998). Reduced coherence in this region may therefore indicate broader inefficiencies in cortical communication and cognitive control. Moreover, Alpha band in Parietal region has been associated by Schulz et al. (2014) (Schulz et al. 2014) to motor excitability, proving that high Alpha coherence in that region is associated with higher MEP amplitudes, in line with our results.

Regarding Beta 1, differences in Parietal and Occipital ROIs are noteworthy findings because they regard specifically regions involved in sensory processing, visuomotor integration, and motor control; additionally, Beta 1 is a frequency band traditionally associated with motor functions. These results, in line with existing literature (Kilner et al. 2000; Pfurtscheller and Lopes da Silva 1999), suggest that, subsequent to the magnetic impulse of TMS, the signal, in the form of Beta 1 waves oscillations, propagates along neural pathways from the stimulation site through Parietal and Occipital regions. Although this phenomenon has been mainly observed in studies employing simultaneous EEG/MEG and TMS recordings, our findings are consistent with this pattern as well. Therefore, to substantiate this hypothesis, a combined TMS‐EEG investigation should be carried out.

This result is further supported by Hordacre et al. (2020), who demonstrated that resting state functional connectivity in the Beta band is higher in stroke patients with preserved cortico‐spinal tract integrity (MEP+) compared to those without motor evoked potentials (MEP−). In their sample of 36 individuals with chronic stroke, MEP+ patients showed greater interhemispheric connectivity in the sensorimotor regions, suggesting a more functionally organized network in those regions that may support better motor recovery and rehabilitation. Moreover, Beta oscillations in the Occipital region were associated with MEP amplitude by Mäki and Ilmoniemi (2010) (Mäki and Ilmoniemi 2010), concluding that motor system excitability appears to be related to activity in occipital areas at frequency ranges associated with visuomotor processing.

Finally, Klados et al. (2016) conducted an EEG study involving 50 older adults with mild cognitive impairment (MCI), reporting that Beta band coherence reorganizes across Occipital, Parietal, and Temporal cortices in tandem with motor and cognitive training, highlighting the dual role of Beta oscillations in supporting both motor and higher‐order cognitive integration. These findings support the hypothesis that lower motor excitability is not an isolated phenomenon but rather part of a more general pattern of neurophysiological vulnerability, potentially impacting both motor and cognitive systems, as reported also by Sadaghiani and Kleinschmidt (2016) and by Schilberg et al. (2021) (Sadaghiani and Kleinschmidt 2016; Schilberg et al. 2021). Cognitive and motor assessments further supported these hypotheses. While grip strength did not significantly differ between groups, corrected MMSE scores were significantly lower in the L‐MEP group, although largely within normal clinical ranges (mean cMMSE ~29 out of 30). This may suggest a subtle lowering in overall cognitive functions among individuals with lower MEP amplitudes, as reported by Costanzo et al. (2024), who indicates MEP as a neurophysiological biomarker for predicting cognitive state. Notably, the lack of difference in grip strength suggests that early neurophysiological changes may not yet be reflected in overt motor performance. These findings may indicate early differences in brain connectivity that, while not yet associated with observable motor deficits, could reflect individual variability in cortico‐spinal integration.

An additional speculative hypothesis is that reduced cortical connectivity and its association with cognitive decline, observed in the L‐MEP group may, at least in part, reflect impaired sensory afferences, which are commonly altered in aging (Humes and Young 2016; Wettstein et al. 2018). Cortical connectivity strongly relies on the integration of sensory inputs. Thus, age‐related declines of sensory functions could reduce the efficiency of cortical processing and connectivity, thereby contributing both to lower motor excitability and poorer cognitive performance. Although we did not directly assess sensory‐evoked potentials in the present study, this possibility warrants consideration and future investigation.

## 5 Limitations

Despite the fact that TMS and EEG data were not obtained simultaneously, their complementary use proved valuable in capturing both cortical excitability and large‐scale network dynamics, providing insights that would have been missed by only using one technique. The results we presented lend further support to the potential of TMS—resting state EEG metrics as early biomarkers of neurophysiological vulnerability and as tools for guiding personalized interventions in contexts such as aging, rehabilitation, or neuromodulation. Nevertheless, longitudinal studies are warranted to assess the predictive ability of these markers over time and to clarify the directionality and stability of the observed associations.

## 6 Conclusions

Taken together, our results indicate that individuals with reduced resting state functional connectivity, particularly in Alpha 2 and Beta 1 bands across Parietal, Occipital and Temporal regions (areas involved in cognitive control, sensory integration, and motor coordination) show lower excitability of the primary motor cortex projecting to the spinal motoneurones, that is, lower cortico‐spinal excitability (L‐MEP group). These neurophysiological differences are accompanied by significantly lower cMMSE scores in the L‐MEP group, despite comparable grip strength between groups. This pattern suggests that subtle alterations in brain connectivity may emerge before detectable motor decline, potentially reflecting early signs of reduced neurofunctional efficiency rather than overt cognitive impairment.

## Author Contributions

**Lorenzo Nucci:** software; data curation; investigation, writing – reviewing and editing. **Federico Frasca:** software; data curation; investigation, writing – reviewing and editing. **Chiara Pappalettera:** software; data curation; investigation, writing – reviewing and editing. **Francesca Ginatempo:** data curation; writing – reviewing and editing. **Nicola Loi:** data curation; writing – reviewing and editing. **Lucia Ventura:** data curation; writing – reviewing and editing. **Mohammed Zeroual:** data curation; writing – reviewing and editing. **Paolo Maria Rossini:** writing – reviewing and editing. **Franca Deriu:** original idea, data curation; writing – reviewing and editing. **Fabrizio Vecchio:** original idea, writing – original draft; data curation; Investigation.

## Funding

This work was funded by the European Union—Next Generation EU—PNRR M6C2—Investimento 2.1 Valorizzazione e potenziamento della Ricerca biomedica del SSN “Brain connectivity and complexity parameters to monitor disease progression in dementia patients and antiinflammatory nanotherapeutics in a preclinical model of Alzheimer's disease”‐ PNRR‐MAD‐2022‐12376667.

## Conflicts of Interest

The authors declare no conflicts of interest.

## Data Availability

The data that support the findings of this study are available on request from the corresponding author.

## References

[cph470136-bib-0001] Antal, A. , B. Luber , A. K. Brem , et al. 2022. “Non‐Invasive Brain Stimulation and Neuroenhancement.” Clinical Neurophysiology Practice 7: 146–165. 10.1016/j.cnp.2022.05.002. 35734582 PMC9207555

[cph470136-bib-0002] Aoki, Y. , R. Ishii , R. D. Pascual‐Marqui , et al. 2015. “Detection of EEG‐Resting State Independent Networks by eLORETA‐ICA Method.” Frontiers in Human Neuroscience 9: 31. 10.3389/fnhum.2015.00031. 25713521 PMC4322703

[cph470136-bib-0003] Binder, J. R. , and R. H. Desai . 2011. “The Neurobiology of Semantic Memory.” Trends in Cognitive Sciences 15, no. 11: 527–536. 10.1016/j.tics.2011.10.001. 22001867 PMC3350748

[cph470136-bib-0004] Breveglieri, R. , S. Borgomaneri , M. Filippini , M. De Vitis , A. Tessari , and P. Fattori . 2021. “Functional Connectivity at Rest Between the Human Medial Posterior Parietal Cortex and the Primary Motor Cortex Detected by Paired‐Pulse Transcranial Magnetic Stimulation.” Brain Sciences 11, no. 10: 1357. 10.3390/brainsci11101357. 34679421 PMC8534070

[cph470136-bib-0005] Buss, S. S. , P. J. Fried , J. Macone , et al. 2023. “Greater Cognitive Reserve Is Related to Lower Cortical Excitability in Healthy Cognitive Aging, but Not in Early Clinical Alzheimer's Disease.” Frontiers in Human Neuroscience 17: 1193407. 10.3389/fnhum.2023.1193407. 37576473 PMC10413110

[cph470136-bib-0006] Buysse, D. J. , C. F. Reynolds , T. H. Monk , S. R. Berman , and D. J. Kupfer . 1989. “The Pittsburgh Sleep Quality Index: A New Instrument for Psychiatric Practice and Research.” Psychiatry Research 28, no. 2: 193–213. 10.1016/0165-1781(89)90047-4. 2748771

[cph470136-bib-0007] Cacciotti, A. , C. Pappalettera , F. Miraglia , et al. 2024. “From Data to Decisions: AI and Functional Connectivity for Diagnosis, Prognosis, and Recovery Prediction in Stroke.” Geroscience 47: 977–992. 10.1007/s11357-024-01301-1. 39090502 PMC11872844

[cph470136-bib-0008] Cárdenas‐Morales, L. , L. J. Volz , J. Michely , et al. 2014. “Network Connectivity and Individual Responses to Brain Stimulation in the Human Motor System.” Cerebral Cortex 24, no. 7: 1697–1707. 10.1093/cercor/bht023. 23395849

[cph470136-bib-0009] Chung, J. W. , E. Ofori , G. Misra , C. W. Hess , and D. E. Vaillancourt . 2017. “Beta‐Band Activity and Connectivity in Sensorimotor and Parietal Cortex Are Important for Accurate Motor Performance.” NeuroImage 144: 164–173. 10.1016/j.neuroimage.2016.10.008. 27746389 PMC5183516

[cph470136-bib-0010] Costanzo, M. , C. Cutrona , G. Leodori , et al. 2024. “Exploring Easily Accessible Neurophysiological Biomarkers for Predicting Alzheimer's Disease Progression: A Systematic Review.” Alzheimer's Research & Therapy 16, no. 1: 244. 10.1186/s13195-024-01607-4. PMC11533378 39497149

[cph470136-bib-0011] Deriu, F. , G. Martinez , N. Loi , et al. 2021. “Reporting Quality of TMS Studies in Neurological Conditions: A Critical Appraisal of the Main Gaps, Challenges and Clinical Implications.” Journal of Neuroscience Methods 362: 109293. 10.1016/j.jneumeth.2021.109293. 34293408

[cph470136-bib-0012] Eswari, B. , S. Balasubramanian , and S. K. M. Varadhan . 2025. “Grasp‐Dependent Modulations in EEG‐EMG Coherence Are Observed in Young but Not Older Adults.” IEEE Transactions on Neural Systems and Rehabilitation Engineering 33: 2025–2033. 10.1109/TNSRE.2025.3569859. 40366843

[cph470136-bib-0013] Ferreri, F. , F. Vecchio , A. Guerra , et al. 2017. “Age Related Differences in Functional Synchronization of EEG Activity as Evaluated by Means of TMS‐EEG Coregistrations.” Neuroscience Letters 647: 141–146. 10.1016/j.neulet.2017.03.021. 28323091

[cph470136-bib-0014] Ferreri, F. , F. Vecchio , D. Ponzo , P. Pasqualetti , and P. M. Rossini . 2014. “Time‐Varying Coupling of EEG Oscillations Predicts Excitability Fluctuations in the Primary Motor Cortex as Reflected by Motor Evoked Potentials Amplitude: An EEG‐TMS Study.” Human Brain Mapping 35, no. 5: 1969–1980. 10.1002/hbm.22306. 23868714 PMC6869650

[cph470136-bib-0015] Fischer, M. H. F. , I. C. Zibrandtsen , P. Høgh , and C. S. Musaeus . 2023. “Systematic Review of EEG Coherence in Alzheimer's Disease.” Journal of Alzheimer's Disease 91, no. 4: 1261–1272. 10.3233/JAD-220508. 36641665

[cph470136-bib-0016] Ginatempo, F. , N. Loi , A. Manca , J. C. Rothwell , and F. Deriu . 2022. “Is It Possible to Compare Inhibitory and Excitatory Intracortical Circuits in Face and Hand Primary Motor Cortex?” Journal of Physiology 600, no. 15: 3567–3583. 10.1113/JP283137. 35801987 PMC9544430

[cph470136-bib-0017] Goldsworthy, M. R. , B. Hordacre , J. C. Rothwell , and M. C. Ridding . 2021. “Effects of rTMS on the Brain: Is There Value in Variability?” Cortex 139: 43–59. 10.1016/j.cortex.2021.02.024. 33827037

[cph470136-bib-0018] Groppa, S. , A. Oliviero , A. Eisen , et al. 2012. “A Practical Guide to Diagnostic Transcranial Magnetic Stimulation: Report of an IFCN Committee.” Clinical Neurophysiology 123, no. 5: 858–882. 10.1016/j.clinph.2012.01.010. 22349304 PMC4890546

[cph470136-bib-0019] Hordacre, B. , M. R. Goldsworthy , E. Welsby , L. Graetz , S. Ballinger , and S. Hillier . 2020. “Resting State Functional Connectivity Is Associated With Motor Pathway Integrity and Upper‐Limb Behavior in Chronic Stroke.” Neurorehabilitation and Neural Repair 34, no. 6: 547–557. 10.1177/1545968320921824. 32436426

[cph470136-bib-0020] Humes, L. E. , and L. A. Young . 2016. “Sensory‐Cognitive Interactions in Older Adults.” Ear and Hearing 37, no. 1: 52S–61S. 10.1097/AUD.0000000000000303. 27355770 PMC4930008

[cph470136-bib-0021] Kauv, P. , M. A. Chalah , A. Créange , J. P. Lefaucheur , J. Hodel , and S. S. Ayache . 2025. “The Corticospinal Tract in Multiple Sclerosis: Correlation Between Cortical Excitability and Magnetic Resonance Imaging Measures.” Journal of Neural Transmission (Vienna) 132, no. 2: 265–273. 10.1007/s00702-024-02849-0. PMC11785694 39417879

[cph470136-bib-0022] Kilner, J. M. , S. N. Baker , S. Salenius , R. Hari , and R. N. Lemon . 2000. “Human Cortical Muscle Coherence Is Directly Related to Specific Motor Parameters.” Journal of Neuroscience 20, no. 23: 8838–8845. 10.1523/JNEUROSCI.20-23-08838.2000. 11102492 PMC6773054

[cph470136-bib-0023] Kim, J.‐S. , H.‐J. Kim , M. Kim , and S.‐H. Kim . 2025. “Hand Motion Control Ability Between Young and Older Adults: Comparative Study.” JMIR Formative Research 9: e65224. 10.2196/65224. 40690275 PMC12322605

[cph470136-bib-0024] Klados, M. A. , C. Styliadis , C. A. Frantzidis , E. Paraskevopoulos , and P. D. Bamidis . 2016. “Beta‐Band Functional Connectivity Is Reorganized in Mild Cognitive Impairment After Combined Computerized Physical and Cognitive Training.” Frontiers in Neuroscience 10: 55. 10.3389/fnins.2016.00055. 26973445 PMC4770438

[cph470136-bib-0025] Klimesch, W. 2012. “α‐Band Oscillations, Attention, and Controlled Access to Stored Information.” Trends in Cognitive Sciences 16, no. 12: 606–617. 10.1016/j.tics.2012.10.007. 23141428 PMC3507158

[cph470136-bib-0026] Kujirai, T. , M. Sato , J. C. Rothwell , and L. G. Cohen . 1993. “The Effect of Transcranial Magnetic Stimulation on Median Nerve Somatosensory Evoked Potentials.” Electroencephalography and Clinical Neurophysiology 89, no. 4: 227–234. 10.1016/0168-5597(93)90100-4. 7688685

[cph470136-bib-0027] Li, N. , T. W. Chen , Z. V. Guo , C. R. Gerfen , and K. Svoboda . 2015. “A Motor Cortex Circuit for Motor Planning and Movement.” Nature 519, no. 7541: 51–56. 10.1038/nature14178. 25731172

[cph470136-bib-0028] Mäki, H. , and R. J. Ilmoniemi . 2010. “EEG Oscillations and Magnetically Evoked Motor Potentials Reflect Motor System Excitability in Overlapping Neuronal Populations.” Clinical Neurophysiology 121, no. 4: 492–501. 10.1016/j.clinph.2009.11.078. 20093074

[cph470136-bib-0029] Mazzoni, M. , L. Ferroni , L. Lombardi , E. Del Torto , M. Vista , and P. Moretti . 1992. “Mini‐Mental State Examination (MMSE): Sensitivity in an Italian Sample of Patients With Dementia.” Italian Journal of Neurological Sciences 13, no. 4: 323–329. 10.1007/bf02223097. 1601631

[cph470136-bib-0030] McFarland, D. J. , L. A. Miner , T. M. Vaughan , and J. R. Wolpaw . 2000. “Mu and Beta Rhythm Topographies During Motor Imagery and Actual Movements.” Brain Topography 12, no. 3: 177–186. 10.1023/a:1023437823106. 10791681

[cph470136-bib-0031] Mesulam, M. M. 1998. “From Sensation to Cognition.” Brain 121, no. 6: 1013–1052. 10.1093/brain/121.6.1013. 9648540

[cph470136-bib-0032] Musaeus, C. S. , M. S. Nielsen , and P. Høgh . 2019. “Altered Low‐Frequency EEG Connectivity in Mild Cognitive Impairment as a Sign of Clinical Progression.” Journal of Alzheimer's Disease 68, no. 3: 947–960. 10.3233/JAD-181081. 30883355

[cph470136-bib-0033] Nitsche, M. A. , and W. Paulus . 2000. “Excitability Changes Induced in the Human Motor Cortex by Weak Transcranial Direct Current Stimulation.” Journal of Physiology 527, no. 3: 633–639. 10.1111/j.1469-7793.2000.t01-1-00633.x. 10990547 PMC2270099

[cph470136-bib-0034] Núñez, P. , J. Poza , C. Gómez , et al. 2019. “Characterizing the Fluctuations of Dynamic Resting‐State Electrophysiological Functional Connectivity: Reduced Neuronal Coupling Variability in Mild Cognitive Impairment and Dementia due to Alzheimer's Disease.” Journal of Neural Engineering 16, no. 5: 056030. 10.1088/1741-2552/ab234b. 31112938

[cph470136-bib-0035] Oliviero, A. , P. Profice , P. A. Tonali , et al. 2006. “Effects of Aging on Motor Cortex Excitability.” Neuroscience Research 55, no. 1: 74–77. 10.1016/j.neures.2006.02.002. 16584795

[cph470136-bib-0036] Ortu, E. , F. Deriu , A. Suppa , E. Tolu , and J. C. Rothwell . 2008. “Effects of Volitional Contraction on Intracortical Inhibition and Facilitation in the Human Motor Cortex.” Journal of Physiology 586, no. 21: 5147–5159. 10.1113/jphysiol.2008.158956. 18787036 PMC2652145

[cph470136-bib-0037] Pappalettera, C. , A. Cacciotti , L. Nucci , F. Miraglia , P. M. Rossini , and F. Vecchio . 2022. “Approximate Entropy Analysis Across Electroencephalographic Rhythmic Frequency Bands During Physiological Aging of Human Brain.” Geroscience 45: 1131–1145. 36538178 10.1007/s11357-022-00710-4 PMC9886767

[cph470136-bib-0038] Pellicciari, M. C. , D. Brignani , and C. Miniussi . 2013. “Excitability Modulation of the Motor System Induced by Transcranial Direct Current Stimulation: A Multimodal Approach.” NeuroImage 83: 569–580. 10.1016/j.neuroimage.2013.06.076. 23845429

[cph470136-bib-0039] Pfurtscheller, G. , and F. H. Lopes da Silva . 1999. “Event‐Related EEG/MEG Synchronization and Desynchronization: Basic Principles.” Clinical Neurophysiology 110, no. 11: 1842–1857. 10576479 10.1016/s1388-2457(99)00141-8

[cph470136-bib-0040] Pierotti, E. , C. Speranza , L. Cattaneo , and L. Turella . 2025. “Investigating Resting‐State Functional Connectivity of the Human Hand Motor System: An Offline TMS‐fMRI Study.” NeuroImage 314: 121254. 10.1016/j.neuroimage.2025.121254. 40339631

[cph470136-bib-0041] Radüntz, T. , J. Scouten , O. Hochmuth , and B. Meffert . 2017. “Automated EEG Artifact Elimination by Applying Machine Learning Algorithms to ICA‐Based Features.” Journal of Neural Engineering 14, no. 4: 046004. 10.1088/1741-2552/aa69d1. 28497769

[cph470136-bib-0042] Ridding, M. C. , and J. C. Rothwell . 1997. “Stimulus/Response Curves as a Method of Measuring Motor Cortical Excitability in Man.” Electroencephalography and Clinical Neurophysiology 105, no. 5: 340–344. 10.1016/s0924-980x(97)00041-6. 9362997

[cph470136-bib-0043] Rossi, S. , A. Antal , S. Bestmann , et al. 2020. “Safety and Recommendations for TMS Use in Healthy Subjects and Patient Populations, With Updates on Training, Ethical and Regulatory Issues: Expert Guidelines.” Clinical Neurophysiology 132, no. 1: 269–306. 10.1016/j.clinph.2020.10.003. 33243615 PMC9094636

[cph470136-bib-0044] Rossini, P. M. , D. Burke , R. Chen , et al. 2015. “Non‐Invasive Electrical and Magnetic Stimulation of the Brain, Spinal Cord, Roots and Peripheral Nerves: Basic Principles and Procedures for Routine Clinical and Research Application. An Updated Report From an I.F.C.N. Committee.” Clinical Neurophysiology 126, no. 6: 1071–1107. 10.1016/j.clinph.2015.02.001. 25797650 PMC6350257

[cph470136-bib-0045] Rossini, P. M. , M. T. Desiato , and M. D. Caramia . 1992. “Age‐Related Changes of Motor Evoked Potentials in Healthy Humans: Non‐Invasive Evaluation of Central and Peripheral Motor Tracts Excitability and Conductivity.” Brain Research 593, no. 1: 14–19. 10.1016/0006-8993(92)91256-e. 1458317

[cph470136-bib-0046] Ruiz‐Gomez, S. J. , C. Gomez , J. Poza , et al. 2019. “Analysis of Volume Conduction Effects on Different Functional Connectivity Metrics: Application to Alzheimer's Disease EEG Signals.” Annual International Conference of the IEEE Engineering in Medicine and Biology Society 2019: 6434–6437. 10.1109/EMBC.2019.8856548. 31947315

[cph470136-bib-0047] Sadaghiani, S. , and A. Kleinschmidt . 2016. “Brain Networks and α‐Oscillations: Structural and Functional Foundations of Cognitive Control.” Trends in Cognitive Sciences 20, no. 11: 805–817. 10.1016/j.tics.2016.09.004. 27707588

[cph470136-bib-0048] Schilberg, L. , S. Ten Oever , T. Schuhmann , and A. T. Sack . 2021. “Phase and Power Modulations on the Amplitude of TMS‐Induced Motor Evoked Potentials.” PLoS One 16, no. 9: e0255815. 10.1371/journal.pone.0255815. 34529682 PMC8445484

[cph470136-bib-0049] Schulz, H. , T. Übelacker , J. Keil , N. Müller , and N. Weisz . 2014. “Now I Am Ready—Now I Am Not: The Influence of Pre‐TMS Oscillations and Corticomuscular Coherence on Motor‐Evoked Potentials.” Cerebral Cortex 24, no. 7: 1708–1719. 23395847 10.1093/cercor/bht024

[cph470136-bib-0050] Sollmann, N. , L. Bulubas , N. Tanigawa , C. Zimmer , B. Meyer , and S. M. Krieg . 2017. “The Variability of Motor Evoked Potential Latencies in Neurosurgical Motor Mapping by Preoperative Navigated Transcranial Magnetic Stimulation.” BMC Neuroscience 18, no. 1: 5. 10.1186/s12868-016-0321-4. 28049425 PMC5209850

[cph470136-bib-0056] The MathWorks Inc. 2022. MATLAB version: 9.13.0 (R2022b). The MathWorks Inc.

[cph470136-bib-0051] Tosoni, A. , S. Pitzalis , G. Committeri , P. Fattori , C. Galletti , and G. Galati . 2015. “Resting‐State Connectivity and Functional Specialization in Human Medial Parieto‐Occipital Cortex.” Brain Structure and Function 220, no. 6: 3307–3321. 25096286 10.1007/s00429-014-0858-x

[cph470136-bib-0052] Wettstein, M. , H. W. Wahl , and V. Heyl . 2018. “Visual Acuity and Cognition in Older Adults With and Without Hearing Loss: Evidence for Late‐Life Sensory Compensation?” Ear and Hearing 39, no. 4: 746–755. 10.1097/AUD.0000000000000531. 29256920

[cph470136-bib-0053] Yoav Benjamini and Daniel, Y. 2001. “The Control of the False Discovery Rate in Multiple Testing Under Dependency.” Annals of Statistics 29, no. 4: 1165–1188. 10.1214/aos/1013699998.

[cph470136-bib-0054] Zhang, J. , J. Yang , Q. Xu , Y. Xiao , L. Zuo , and E. Cai . 2024. “Effectiveness of Virtual Reality‐Based Rehabilitation on the Upper Extremity Motor Function of Stroke Patients: A Protocol for Systematic Review and Meta‐Analysis.” PLoS One 19, no. 11: e0313296. 10.1371/journal.pone.0313296. 39509415 PMC11542779

[cph470136-bib-0055] Ziemann, U. , S. Lönnecker , B. J. Steinhoff , and W. Paulus . 1996. “Effects of Antiepileptic Drugs on Motor Cortex Excitability in Humans: A Transcranial Magnetic Stimulation Study.” Annals of Neurology 40, no. 3: 367–378. 10.1002/ana.410400306. 8797526

